# Socioeconomic status as a determinant of survival in glioblastoma: a systematic review and meta-analysis

**DOI:** 10.1007/s10143-025-03647-2

**Published:** 2025-06-11

**Authors:** Jeremiah Hilkiah Wijaya, Saad Hulou, Brandon Lucke-Wold, Wiley Braxton Gillam V, Chris B. Lamprecht, FNU Ruchika, Bryce Schneider, Devon Foster, Pouria Abolfazli, Miguel David Quintero-Consuegra, Zahraa F. Al-Sharshahi, Julius July

**Affiliations:** 1 School of Public Health and Preventive Medicine, Monash University, Melbourne, VIC Australia; 2 Department of Neurosurgery, College of Medicine, University of Kentucky, Lexington, KY USA; 3 Department of Neurosurgery, College of Medicine, University of Florida, Gainesville, FL USA; 4 Department of Neurosurgery, Johns Hopkins School of Medicine, Baltimore, MD USA; 5 College of Medicine, Xi’an Jiaotong University, Xi’an, China; 6 Department of Neurosurgery, Cedars-Sinai Medical Center, Los Angeles, CA USA; 7 Department of Neurosurgery, Universitas Pelita Harapan, Tangerang, Banten, Indonesia

**Keywords:** Socioeconomic status, Glioblastoma, Survival, Disparities

## Abstract

Studies highlight the importance of social factors, such as race and socioeconomic status (SES), in disease management. Integrating these factors helps improve understanding of disease outcomes and the development of effective treatments. Thus, we aimed to systematically identify and review relevant studies exploring the relationship between SES and survival outcomes in GBM patients. An extensive exploration of academic databases, including Scopus, EMBASE, and PubMed, was undertaken, covering records from their inception until December 14, 2024. This search targeted specific keywords and their synonymous terms: glioblastoma, survival, and socioeconomic. We included 230,601 patients, with many individuals being diagnosed between the ages of 46 and 65. Notably, Female exhibited a higher risk (HR = 1.07, 95% CI: 1.05–1.09) of death compared to male, while African Americans demonstrated a higher risk than Caucasians (HR = 0.92, 95% CI: 0.88–0.97), alongside Hispanics (HR = 0.85, 95% CI: 0.72–0.99) and other races (HR = 0.78, 95% CI: 0.73–0.85). Similarly, unmarried individuals faced a higher risk (HR = 1.14, 95% CI: 1.09–1.20) compared to married counterparts. Noteworthy trends were observed in insurance, where private payers (HR = 1.11, 95% CI: 1.06–1.15) and government-based insurance (HR = 1.09, 95% CI: 1.00-1.19) showed increased risks compared to private insurance. However, associations in widowhood (HR = 2.45, 95% CI: 0.34–17.40), comorbidities (HR = 1.05, 95% CI: 0.93–1.18), median household income (MHI) (HR = 0.94, 95% CI: 0.85–1.05), and rural living (HR = 1.06, 95% CI: 0.98–1.16) were non-significant or inconclusive. These findings emphasize the complex interplay between socioeconomic factors and health risks, highlighting the necessity for tailored interventions to address health disparities across diverse demographic groups.

**Supplementary Information:**

The online version contains supplementary material available at 10.1007/s10143-025-03647-2.

## Introduction

Research focusing on the influence of socioeconomic factors on glioblastoma outcomes holds paramount importance due to the profound impact these determinants have on disease progression and patient outcomes. The intricate interplay between socioeconomic status (SES) and demographic patient characteristics has been extensively documented, revealing their consequential effects on treatment decisions and subsequent glioblastoma prognoses [6, 22]. Notably, adverse SES conditions have been closely associated with delays in the initiation of radiotherapy and poorer overall survival rates among newly diagnosed glioblastoma patients [12, 19]. 

Several studies have identified a positive association between higher SES and the incidence of glioblastoma multiforme (GBM), the most aggressive form of glioma. For example, Nilsson et al. (2018) reported a 1.14-fold increased risk of developing GBM among individuals in the highest SES quartile compared to those in the lowest [15]. Similarly, Plascak and Fisher (2013) found elevated GBM incidence rates among populations with higher SES [18]. Expanding on these findings, Gorenflo et al. (2023) demonstrated that higher area-level SES was not only linked to increased incidence but also to better survival outcomes in the United States, underscoring socioeconomic disparities in both disease burden and prognosis [7]. These disparities may be partially explained by confounding factors such as earlier diagnosis, access to specialized care, geographic advantages, greater health literacy, stronger psychosocial support, and improved treatment adherence—factors more commonly found among individuals with higher SES [24]. Properly adjusting for these variables is essential to accurately assess the independent effect of SES on GBM outcomes.

Studies highlight the importance of social factors, such as race and SES, in disease management. Integrating these factors helps improve understanding of disease outcomes and the development of effective treatments. By understanding the impact of socioeconomic factors on glioblastoma outcomes, healthcare practitioners can tailor interventions to mitigate disparities and elevate the overall standard of care for individuals grappling with this formidable manifestation of brain cancer. Thus, we aimed to systematically identify and review relevant studies exploring the relationship between SES and survival outcomes in GBM patients.

## Materials and methods

### Search strategy

An extensive exploration of academic databases, including Scopus, EMBASE, and PubMed, was undertaken, covering records from their inception until December 14, 2024. This search targeted specific keywords and their synonymous terms: glioblastoma, survival, and socioeconomic (see Table 1). The systematic review and meta-analysis adhered to the PRISMA guidelines, with rayyan.ai serving as the platform for study selection [16]. 

**Table 1:** Search queries used to retrieve studies from the databases

Database	Search terms
Scopus	( ( ( ( glioblastoma AND multiforme ) OR ( glioblastoma ) ) OR ( high AND grade AND glioma ) ) OR ( grade AND iv AND glioma ) ) AND ( socioeconomic AND status )
EMBASE	((glioblastoma multiforme or Glioblastoma or high grade glioma or grade IV glioma) and socioeconomic status).mp. [mp = title, abstract, heading word, drug trade name, original title, device manufacturer, drug manufacturer, device trade name, keyword heading word, floating subheading word, candidate term word]
PubMed	(“social class“[MeSH Terms] OR (“social“[All Fields] AND “class“[All Fields]) OR “social class“[All Fields] OR (“socioeconomic“[All Fields] AND “status“[All Fields]) OR “socioeconomic status“[All Fields]) AND (“glioblastoma“[MeSH Terms] OR “glioblastoma“[All Fields] OR “glioblastomas“[All Fields] OR (“glioma“[MeSH Terms] OR “glioma“[All Fields] OR (“high“[All Fields] AND “grade“[All Fields] AND “glioma“[All Fields]) OR “high grade glioma“[All Fields]) OR ((“grade“[All Fields] OR “graded“[All Fields] OR “grades“[All Fields] OR “grading“[All Fields] OR “gradings“[All Fields]) AND (“ieee intell veh symp“[Journal] OR “iv“[All Fields]) AND (“glioma“[MeSH Terms] OR “glioma“[All Fields] OR “gliomas“[All Fields] OR “glioma s“[All Fields])))

### Article selection process

Peer-reviewed studies examining SES as an independent variable influencing access to GBM care were included. Two independent reviewers (JH and SH) screened titles and abstracts for eligibility, followed by full-text screening of potentially relevant studies. Discrepancies were resolved by a third reviewer (JJ).

### Eligibility criteria

Eligible studies were required to report survival data and include demographic information such as age, sex, ethnicity, and relevant socioeconomic indicators. We considered a range of study designs, including cohort, case-control, and cross-sectional studies, without restriction on year of publication. However, to ensure methodological rigor and the inclusion of primary data, we excluded case reports, reviews, editorials, commentaries, and letters without original research data. Only articles written in English or with an English translation were included.

### Outcome of interests

The primary outcome of interest was overall survival (OS), assessed across various socioeconomic factors. These included sex (male vs. female), race (African American, Caucasian, Hispanic, and others), marital status (married, unmarried, widowed, or unknown), insurance type (private vs. government-based), and presence of comorbidities. Median household income (MHI) was analyzed by comparing lower- and higher-income brackets, and area of residence was assessed by contrasting urban and metropolitan settings. Because definitions of MHI and geographic classifications varied across studies, we did not impose a uniform cutoff. Instead, we harmonized data by categorizing income and geographic status using relative measures (e.g., tertiles or standardized classifications) based on each study’s definitions, ensuring comparability across studies.

### Data extraction

For this systematic review and meta-analysis, the following information was extracted from each article: the title, link, source (including the database), year of publication, authors, country of study, journal name, study type/design, sample size, sampling method, inclusion and exclusion criteria, research question and primary aims, intervention details, classification of SES, the independent and dependent variables, confounders, population demographics (age, gender, sex, income, education, race/ethnicity, insurance coverage/type, comorbidities), primary outcome and its measurement, secondary outcomes and their measurement, period/time (duration and years), main results/conclusion, and GBM management details.

### Quality assessment

In the assessment of observational studies’ quality, the researchers utilized the Risk of Bias in Non-randomized Studies of Interventions (ROBINS-I) tool. This tool served as a methodological instrument for critically appraising the risk of bias in non-randomized studies, offering a structured framework for evaluating various domains of study design, conduct, and analysis. Through the ROBINS-I assessment, potential sources of bias, including confounding, selection bias, misclassification, and measurement error, among others, were systematically identified. By employing this tool, the researchers enhanced the validity and reliability of their assessments of observational studies, thereby ensuring the robustness and integrity of the evidence synthesized in the systematic review and meta-analysis.

### Data analysis

The analysis utilized RStudio version 0.97.551, developed by RStudio, Inc. The metafor function package was employed to create graphics and perform quantitative measurements in this analysis. The pooled effect estimates were determined using hazard ratio (HR) and 95% confidence interval (CI). Heterogeneity across studies was assessed using the inconsistency index (I2), which ranged from 0 to 100%. An I2 value greater than 50% or a p-value less than 0.10 indicated statistically significant heterogeneity. The inverse-variance method with a random-effects model was employed for HR meta-analysis, regardless of the presence of heterogeneity. Publication bias was qualitatively assessed using funnel plot analysis, supplemented by a regression-based Egger’s test to evaluate the potential for small-study effects. Two-tailed p-values were used in the study, with statistical significance set at *p* < 0.05, except for heterogeneity where *p* < 0.10 was considered significant.

## Results

**Fig. 1:**
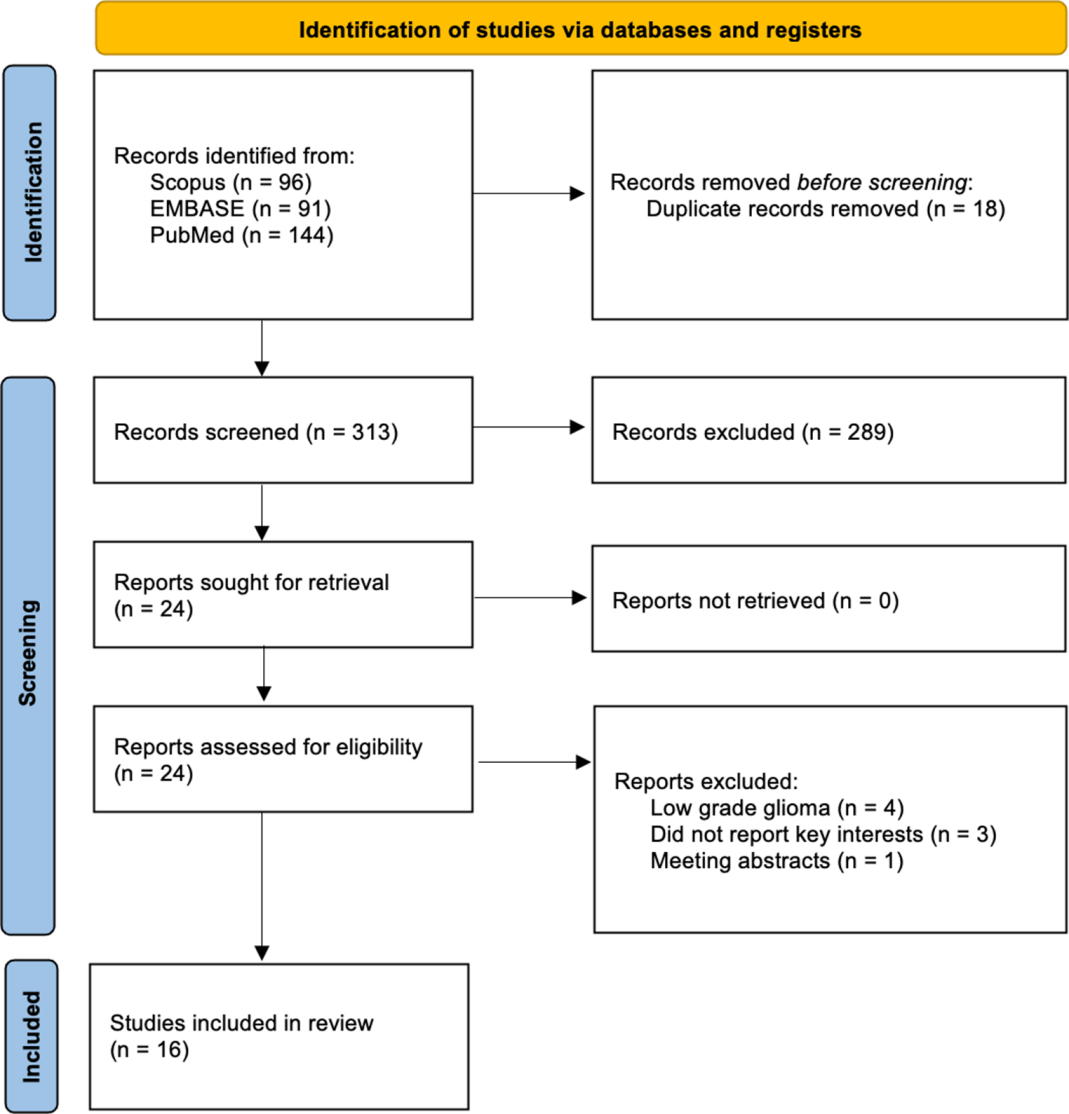
PRISMA diagram for study screening

The PRISMA 2020 flow diagram (Fig. 1) outlines the systematic review process, focusing on searches conducted solely within databases and registers. In the first phase, a total of 331 records were identified from Scopus (*n* = 96), EMBASE (*n* = 91), and PubMed (*n* = 144). After removing 18 duplicate records, 313 unique records remained for screening. In the subsequent phase, Study Screening, 289 records were excluded based on title and abstract review. Of the remaining 24 reports, all were successfully retrieved and assessed for eligibility. Following full-text evaluation, 16 studies met the inclusion criteria and were included in the final review [1–3, 6, 9–14, 17, 19, 20, 22, 26]. The reasons for exclusion during this phase included four studies on low-grade glioma, two studies that did not report key interests, and one meeting abstract.

The aggregated dataset encompasses a comprehensive analysis of demographic, clinical, and socioeconomic variables across multiple studies, with a cumulative patient population of 230,601 individuals from the USA, China, Italy, Brazil, Sweden, and the Philippines. The treatment modalities employed demonstrate significant variability, reflecting the heterogeneity of clinical decision-making. Specifically, biopsy was utilized in 18,812 cases, while surgical interventions were categorized into gross total resection (GTR), performed in 25,298 cases, and subtotal resection (STR), conducted in 22,352 cases. Additionally, a conservative management approach was adopted for 17,499 patients.

Concerning the total number of patients who received either radiation therapy alone, chemotherapy alone, or a combination of both, the data cannot be aggregated by the researcher due to some studies reporting overlapping information across groups. Nonetheless, the overall figures indicate that 118,188 patients underwent radiation therapy, 19,906 received chemotherapy, and 78,899 were treated with a combination of radiation and chemotherapy. Tumor localization data revealed a predominance of supratentorial tumors, accounting for 47,168 cases, alongside infratentorial tumors (*n* = 566) and overlapping tumor regions (*n* = 9,200). Laterality was also documented, with left-sided tumors (*n* = 453), right-sided tumors (*n* = 453), and bilateral tumors (*n* = 90) identified.

From a demographic perspective, the racial and ethnic composition of the cohort highlights substantial diversity. The majority of participants identified as Caucasian (*n* = 182,835), followed by African American (*n* = 11,563), Hispanic (*n* = 9,343), Asian (*n* = 3,749), and other racial or ethnic categories, which collectively accounted for 23,111 individuals. Insurance coverage patterns further illustrate disparities in healthcare access, with private insurance being the most prevalent form of coverage, utilized by 88,796 patients, while government-based insurance supported 61,407 individuals. Geographic distribution data revealed that the majority of patients resided in metropolitan areas (*n* = 85,804), with smaller proportions inhabiting urban areas (*n* = 34,275) and rural areas (*n* = 1,792). The categorization of living area and marital status deviates from conventional classifications such as rural, metropolitan, or urban designations, adopting instead alternative frameworks tailored to the specific contexts of each study. For instance, geographic distribution is delineated using region-specific criteria. In studies conducted in the United States, populations are classified by state, while in Italy, the population is segmented into three distinct regions—south, central, and north—reflecting regional distinctions unique to the country’s socio-geographic structure.

Marital status demonstrated that the majority of patients were married (*n* = 47,563), followed by those who were single (*n* = 7,360), widowed (*n* = 5,010), or had an unknown marital status (*n* = 892). It is important to note that marital status is operationalized differently across studies, with some investigations distinguishing between categories such as single, unmarried, and widowed, despite these groups sharing the common characteristic of being unpartnered. In light of these variations, the classifications are presented in their original form as reported by the respective studies. Additional details regarding patient characteristics are provided in Tables 2 and 3 for further reference and analysis.

**Table 2:** Demographic attributes derived from incorporated studies investigating the influence of socioeconomic status on glioblastoma survival

Study ID	Country	Data collection	Total cohort, *n*	Treatment received	Tumor details
Pollom 2018	USA	2010–2013	12,738	Biopsy = 2768 STR = 4700 GTR = 5270 Radiation and Chemotherapy = 12,738	n/r
Liu 2020	USA	2005–2016	28,952	Radiation = 21,690 Chemotherapy = 19,906 Surgery = 5516	**Location** Supratentorial = 22,630 Infratentorial = 365 Overlapping = 5957
Tosoni 2021	Italy	April 2017– December 2017	106	Biopsy = 8 STR = 74 GTR = 24	**MGMT methylation** Methylated = 47 Unmethylated = 54
Estevez-Ordonez 2023	USA	2008–2019	995	Biopsy = 298 STR = 250 GTR– 447 Chemotherapy = 127 Radiation and Chemotherapy = 868	**Location** Supratentorial = 853 Infratentorial = 47 Other = 86 **Laterality** Right = 432 Left.= 447 Bilateral = 86
Xie 2018	USA	2004–2015	30,767	Surgery = 22,835 Conservative = 7841 Unknown = 91	**Laterality** Unilateral = 50,178 Bilateral = 10,768 Paired = 568
Chang 2005	USA	1988–2001	10,987	Biopsy = 2510 Surgery = 8071 Conservative = 71 Radiation = 8127	**Location** Supratentorial = 10,987 **Site** Frontal = 2521 Temporal = 2538 Parietal = 1887 Occipital = 474 Others = 3567
Rong 2016	USA	2007–2012	16,690	Surgery = 10,382 Radiation = 8163	**Tumor size** ≤ 45 mm = 5891 > 45 mm = 5598 Unknown = 2176
Lynch 2012	Brazil	1998–2008	66	STR = 28 GTR = 38 Radiation = 45	n/r
Barnholtz-Sloan 2007	USA	June 1991– December 1999	1530	Biopsy = 145 Surgery = 318 Multiple treatment = 1067	**Location** Supratentorial = 1073 Other = 110 Overlapping = 347
Hong 2022	Philippines	2015–2019	48	Biopsy = 5 STR = 27 GTR = 16 Radiation and chemotherapy = 7 Radiation = 11	**Laterality** Right = 21 Left = 23 Bilateral = 4 **Location** Frontal = 14 Parietal = 5 Temporal = 6 ≥ 2 lobe = 19 Multifocal = 4
Hodges 2021	USA	2004–2014	103,652	Biopsy = 9306 GTR = 12,448 STR = 10,297 Unknown = 52,599 Conservative = 8827 Radiation = 65,128 Chemotherapy = 59,100	**Focality** Unifocal = 35,521 Multifocal = 7562 Unknown = 53,394 n/r
Marta 2021	Brazil	1999–2000	4511	Surgery = 622 Radiation = 162 Chemotherapy = 29 Surgery + Radiation = 319 Surgery + Chemotherapy = 57 Radiation + Chemotherapy = 158 Surgery + Radiation + Chemotherapy = 698 Other = 141 Conservative = 202	n/r
Bohn 2018	USA	2010–2014	3517	GTR = 1253 STR = 1657 Conservative = 558	**Location** Frontal = 1328 Temporal = 1201 Parietal = 747 Occipital = 197
Pan 2015	USA	2000–2009	14,675	Biopsy = 3728 STR = 5185 GTR = 5762 Radiation = 14,675	**Location** Supratentorial = 11,625 Infratentorial = 154 Overlapping = 2896
Bergqvist 2018	Sweden	2001–2013	1149	Surgery + radiotherapy = 1149	
Kasl 2016	USA	2000–2014	218	Biopsy = 44 STR = 134 GTR = 40 Radiation = 187 Chemotherapy = 171	**Focality** Unifocal = 184 Multifocal = 34

*GTR: gross total resection; MGMT: O6-methylguanine-DNA methyltransferase; n: number; n/r: not reported; STR: subtotal resection; USA: United States of America

**Table 3:** Demographic overview categorized by socioeconomic status

Study ID	Race	Household income	Insurance	Area of living	Marital status
Pollom 2018	Caucasian = 11,634; African American = 640; Other = 464	<$48,000 = 4,284; >$48,000 = 8,324; Unknown = 130	Private = 6858 Government-based = 5743 Unknown = 137	Metropolitan = 10,222 Urban = 20,179 Unknown = 437	-
Liu 2020	Caucasian = 23,101 Hispanic = 2872 African American = 1702 Asian = 1277	<$40,000 = 1388 $40,000–$60,000 = 8644 $60,000–$80,000 = 11,971 $80,000–$100,000 = 5552 >$100,000 = 1397	Private = 23,573 Not Insured = 760 Unknown = 4619	-	-
Tosoni 2021	-	< 36,152€ = 35 36,153 − 70,000€ = 44 70,001-100,000€ = 7 > 100,000€ = 20	Government-based = 106	North Italy = 76 Central Italy = 16 South Italy = 14	-
Estevez-Ordonez 2023	Caucasian = 857 African American = 117 Others = 21	Low = 294 Middle = 691 High = 10	Private = 686, Government-based = 273 Uninsured = 36	Metropolitan = 719 Micropolitan = 151 Rural = 38 Small town = 87	Married = 697 Widowed = 120 Single = 128 Unknown = 53
Xie 2018	Caucasian = 27,557 African American = 1692 Others = 1441 Unknown = 57	Quartile 1 = 7685 Quartile 2 = 7663 Quartile 3 = 7705 Quartile 4 = 7712	Insured = 20,059 Government-based = 2453 Uninsured = 754 Unknown = 7501	Northeast = 5028 South = 6472 North Central = 2996 West = 16,271	Married = 20,076 Divorced = 2872 Widowed = 3550 Single = 4269
Chang 2005	Caucasian = 10,032 African American = 486 Others = 454 Unknown = 15	-	-	-	Married = 7396 Divorced = 888 Widowed = 1339 Single = 1076 Seperated = 48
Rong 2016	African American = 806 Caucasian = 12,124 Other = 695 Unknown = 40	-	Private = 11,591 Government-based = 1516 Uninsured = 558	Alaska = 8 California = 5654 Connecticut = 701 Georgia = 1383 Hawaii = 113 Iowa = 620 Kentucky = 830 Louisiana = 651 Michigan = 701 New Jersey1505 New Mexico = 244 Utah = 377 Washington = 878	Married = 8613 Not married = 4607 Unknown = 445
Lynch 2012		Low income = 66	Government-based = 66	-	-
Barnholtz-Sloan 2007	Caucasian = 645 Hispanic = 53 African American = 18 Asian = 25	≤ $30,000 = 427 > $30,000 = 1084	Government-based = 1530	North East = 282 South = 86 Midwest = 499 West = 663	Married = 980 Not married = 529
Hong 2022	-	Low = 8 Lower middle = 40	Government-based = 48	Urban = 21 Rural = 27	Married = 35 Widowed = 1 Single = 12
Hodges 2021	Caucasian = 81,900 Hispanic = 4815 Asian = 1638 African American = 5124	-	Private = 40,523 Government-based = 47,244 Uninsured = 3433 Unknown = 2277	Metropolitan = 73,567 Urban = 14,075 Rural = 1727 Unknown = 4108	-
Marta 2021		-	Private = 2436 Government-based = 2075	Metropolitan = 1296 Others = 3215	-
Bohn 2018	Caucasian = 2890 Hispanic = 192 Asian = 186 African American = 205	-	Private = 3029 Government-based = 283 Not insured = 97	-	-
Pan 2015	Caucasian = 11,881 African American = 773 Asian = 623 Hispanic = 1411	-	-	Northeast = 2561 Midwest = 1521 South = 2943 West = 7650	Married = 9707 Single = 1875 Unmarried = 2699 Unknown = 394
Bergqvist 2018	-	Low = 430 Middle = 478 Higher = 220	-	-	-
Kasl 2016	Caucasian = 205 Others = 13	Low = 65 Middle = 125 High = 28	Private = 100 Government-based = 118	-	Married = 59 Not married = 159

According to the meta-analysis, females exhibited a higher risk (HR = 1.07, 95% CI: 1.05–1.09, *p* < 0.00001) of death compared to the males counterpart, while African Americans demonstrated a higher risk than Caucasians (HR = 0.92, 95% CI: 0.88–0.97, *p* = 0.0004), alongside Hispanics (HR = 0.85, 95% CI: 0.72–0.99, *p* = 0.04) and other races (HR = 0.78, 95% CI: 0.73–0.85, *p* < 0.00001). Similarly, unmarried individuals faced a higher risk (HR = 1.14, 95% CI: 1.09–1.20, *p* < 0.00001) compared to married counterparts. Noteworthy trends were observed in insurance, where private payers (HR = 1.11, 95% CI: 1.06–1.15, *p* < 0.00001) and government-based insurance (HR = 1.09, 95% CI: 1.00-1.19, *p* = 0.05) showed increased risks compared to private insurance. However, associations in widowhood (HR = 2.45, 95% CI: 0.34–17.40, *p* = 0.37), comorbidities (HR = 1.05, 95% CI: 0.93–1.18, *p* = 0.43), MHI (HR = 0.94, 95% CI: 0.85–1.05, *p* = 0.28), and rural living (HR = 1.06, 95% CI: 0.98–1.16, *p* = 0.16) were non-significant or inconclusive. Summary of meta-analysis was displayed in Table 4.

**Table 4:** The analysis of socioeconomic variables and their association with risk factors

Socioeconomic variables		Reference	HR [95% CI]	I^2^	*p*-value
**Sex**	Male	Female	1.07 [1.05, 1.09]	9%	< 0.00001
**Race**	African American	Caucasian	0.92 [0.88, 0.97]	47%	0.0004
	Hispanic	Caucasian	0.85 [0.72, 0.99]	95%	0.04
	Others	Caucasian	0.78 [0.73, 0.85]	68%	< 0.00001
**Marital status**	Unmarried	Married	1.14 [1.09, 1.20]	31%	< 0.00001
	Widow	Married	2.45 [0.34, 17.40]	70%	0.37
	Unknown	Not married	0.87 [0.75, 1.01]	63%	0.07
**Insurance**	Private payer	Private insurance	1.11 [1.06, 1.15]	0%	< 0.00001
	Government based insurance	Private insurance	1.09 [1, 1.19]	66%	0.05
**Comorbid**	With comorbid	No comorbid	1.05 [0.93, 1.18]	41%	0.43
**MHI**	Low income	High income	0.94 [0.85, 1.05]	72%	0.28
**Area of living**	Urban	Metropolitan	1.07 [1.04, 1.10]	0%	< 0.00001
	Rural	Metropolitan	1.06 [0.98, 1.16]	0%	0.16

**Fig. 2:**
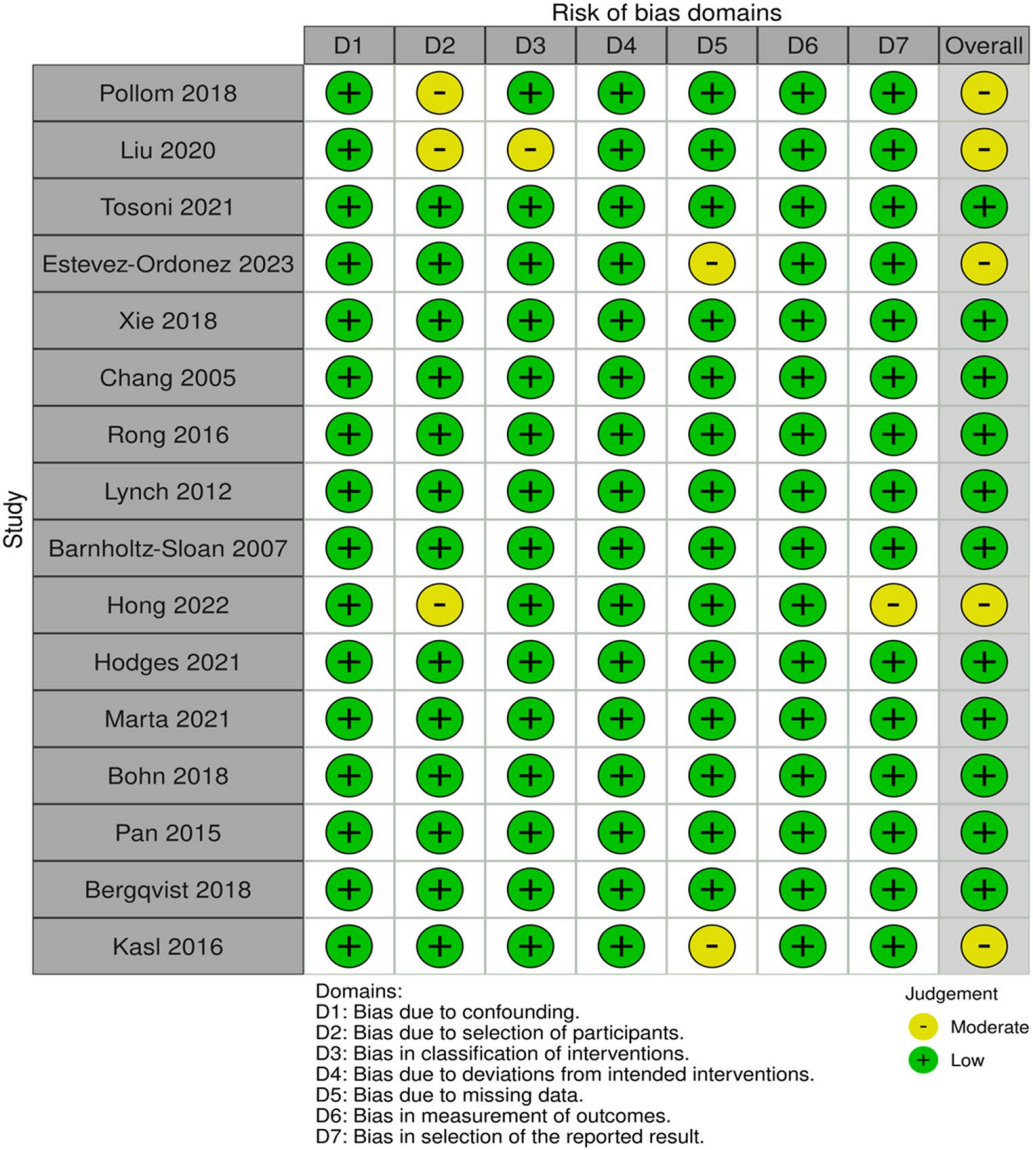
ROBINS-I for study assessment

The quality assessment using the ROBINS-I indicated a low-to-moderate risk of bias (Fig. 2). An asymmetrical distribution was observed for sex and MHI funnel plot, which analyzed the impact of those variables on survival outcomes in GBM patients (supplementary files).

## Discussion

The present study’s findings on socioeconomic variables and their association with health risk factors align in part with existing literature yet al.so introduce nuanced perspectives that warrant further exploration. For instance, the observation that lower MHI does not significantly alter risk (HR = 0.94 [0.85, 1.05], *p* = 0.28) contrasts with a substantial body of research underscoring the adverse health impacts of low socioeconomic status [6, 10]. This discrepancy might be attributed to the diverse composition of the study sample or the robust adjustment for confounders such as insurance type and race/ethnicity, which could mitigate the apparent impact of income. Similarly, the finding that racial or ethnic minorities exhibit lower HR compared to Caucasians diverges from conventional narratives about minority health disparities [27]. The adjusted model possibly accounts for overlapping disadvantages, thus unveiling a more complex relationship between race and health outcomes than previously understood.

Gender differences in health risks, highlighted by the elevated hazard ratio for males (HR = 1.07 [1.05, 1.09]), corroborate prior studies indicating increased vulnerabilities among men due to behavioral and social factors. This consistency across diverse populations affirms the significance of gender as a determinant of health, suggesting targeted interventions are necessary. Men may be less likely to seek timely medical attention, adhere to treatment protocols, or access supportive care—factors that are further exacerbated by SES-related barriers such as limited healthcare resources or financial strain [4, 25]. 

Meanwhile, marital status emerges as another critical factor, with unmarried individuals facing higher risks (HR = 1.14 [1.09, 1.20])—a trend supported by literature linking marriage to better health through mechanisms like social support. Married individuals are more likely to benefit from emotional encouragement, assistance with medical decision-making, and help navigating the healthcare system. These forms of support can lead to earlier diagnosis, greater treatment adherence, and improved overall outcomes [4, 25]. However, the wide CI observed for widows’ points to unique challenges within this subgroup, urging deeper investigation into how different marital transitions affect health risks [4, 25]. 

Insurance type and area of living further illustrate the intricate dynamics between access to resources and health outcomes. The modestly increased risks associated with private payer and government-based insurance relative to private insurance resonate with discussions on healthcare accessibility and quality, as patients with limited or less comprehensive insurance may face barriers such as delayed diagnosis, restricted treatment options, or reduced access to specialized care [5, 21]. Urban living presents a mixed picture; while urban residents face slightly elevated risks (HR = 1.07 [1.04, 1.10]), rural-urban differences appear less pronounced in this analysis, likely due to adjustments for socioeconomic factors. These synthesized insights highlight the layered interactions of demographic, socioeconomic, and environmental elements in shaping health outcomes [23]. Emphasizing diversity and intersectionality, the study enriches our understanding of health disparities and calls for tailored strategies addressing these multifaceted influences.

### Study limitations and suggestion for future studies

A key limitation of this study is that, with fewer than 10 studies included in the meta-analysis, a meta-regression could not be performed, and even if attempted, the results would likely be insignificant due to insufficient data; therefore, future studies with larger sample sizes are encouraged to validate these findings. Secondly, the high heterogeneity observed across included analyses, largely due to variability in study designs, population characteristics, and definitions of socioeconomic variables. Additionally, comparing outcomes between high-income countries and low- and middle-income countries could provide valuable insights and should be considered in future research to address gaps in understanding disparities across diverse economic contexts. The disparities in conditions like GBM among racial and ethnic groups may stem not only from race itself but also from overlapping factors such as insurance coverage, income levels, and systemic inequities [8]. However, inconsistencies in categorizing variables like MHI—with differing income brackets preventing a comprehensive meta-analysis—introduce ambiguity into the results. Despite these challenges, the study included MHI findings, potentially complicating the interpretation of socioeconomic impacts on health outcomes.

For stakeholders and policymakers, addressing these disparities requires a multifaceted approach that considers intersecting socioeconomic, demographic, and geographic factors while acknowledging the study’s limitations. Broad policy initiatives, such as expanding insurance coverage, subsidizing healthcare for low-income households, and investing in preventive care and community-based support systems, can help mitigate systemic barriers faced by underserved populations. Policymakers should also prioritize comprehensive data collection efforts that account for intersectional identities and underrepresented groups, enabling more precise identification of at-risk populations.

## Conclusions

In conclusion, our study explored socioeconomic variables’ connections to risk factors. Females exhibited slightly higher risks of death than males, while Caucasians had lower risks than African Americans, with Hispanics and other groups also showing decreased risks. Marital status notably impacted risk, with unmarried and widowed individuals at higher risk. Both private and government-based insurance correlated with increased risks. However, comorbidities and MHI had minimal effects on risk levels. These findings emphasize the complex interplay between socioeconomic factors and health risks, highlighting the necessity for tailored interventions to address health disparities across diverse demographic groups.

## Electronic supplementary material

Below is the link to the electronic supplementary material.

Supplementary Material 1

## Data Availability

The datasets generated during and/or analysed during the current study are available from the corresponding author on reasonable request.
