# Supplementary material for: Socioeconomic status as a determinant of survival in glioblastoma: a systematic review and meta-analysis
Source: Neurosurg Rev. 2025 Jun 11;48(1):500. doi: 10.1007/s10143-025-03647-2 (PMC12152019; doi:10.1007/s10143-025-03647-2)

**Supplementary file 1.** A meta-analysis investigating how sex affects the overall survival of patients diagnosed with GBM.


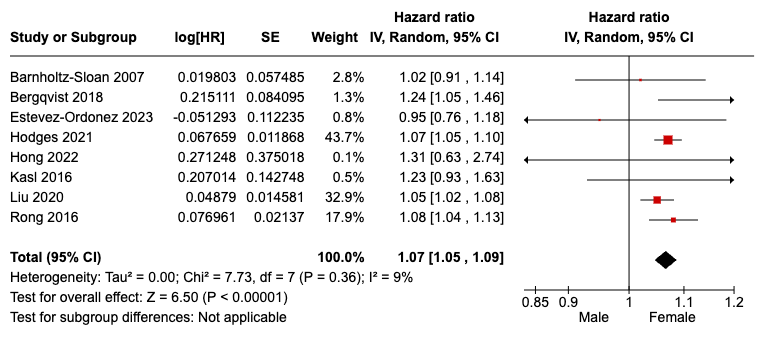

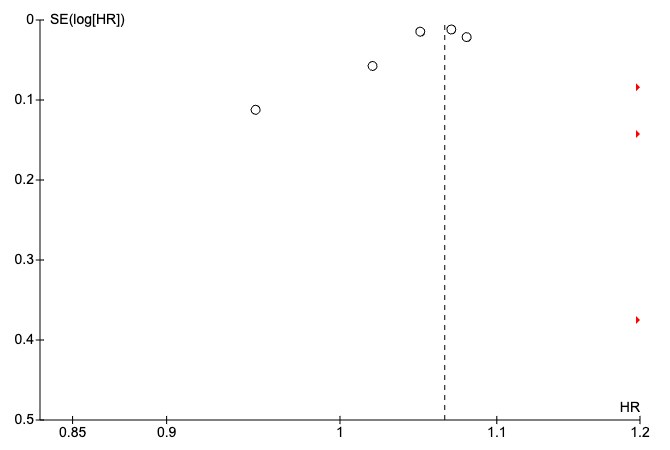


**Supplementary file 2.** A meta-analysis investigating African American vs Caucasians affects the overall survival of patients diagnosed with GBM.


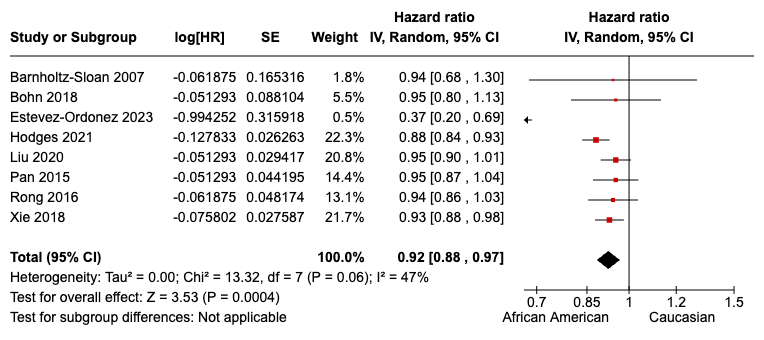

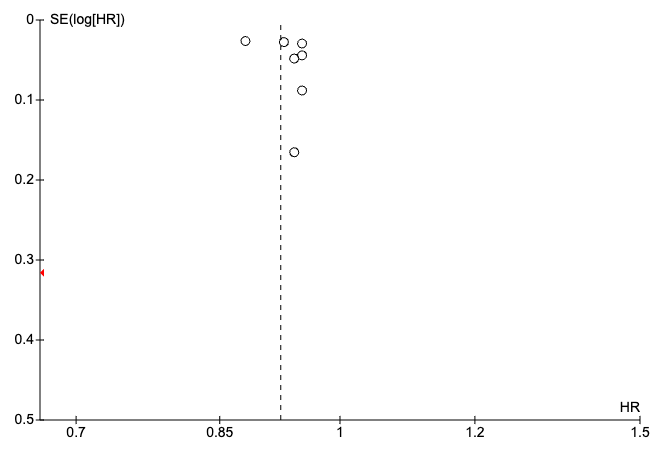


**Supplementary file 3.** A meta-analysis investigating Hispanic vs Caucasians affects the overall survival of patients diagnosed with GBM.


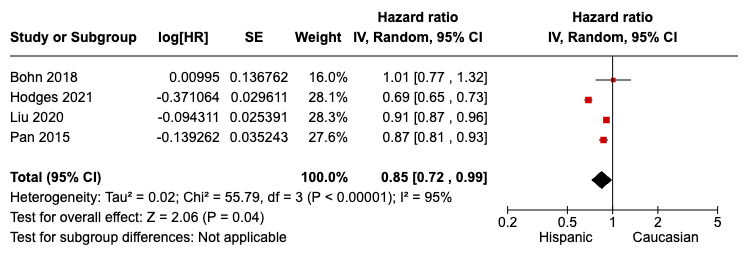


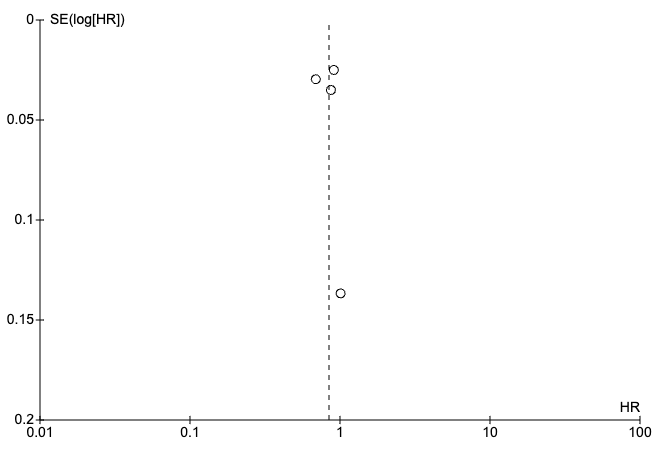


**Supplementary file 4.** A meta-analysis investigating other race vs Caucasians affects the overall survival of patients diagnosed with GBM.


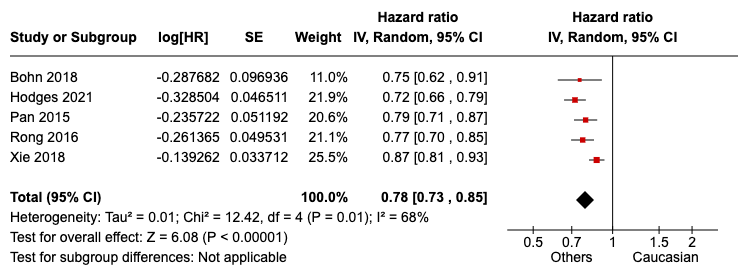

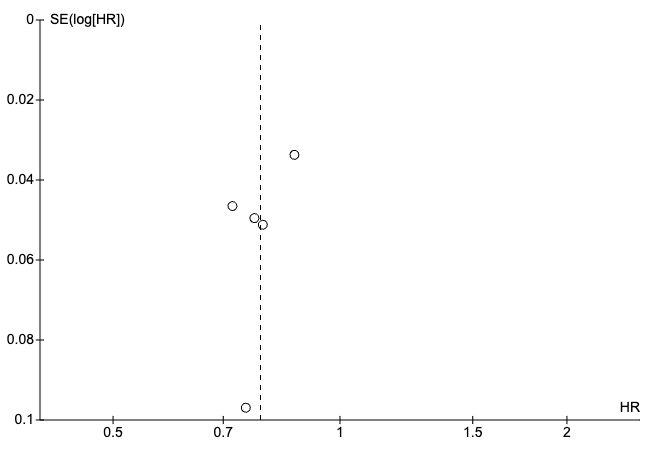


**Supplementary file 5.** A meta-analysis investigating unmarried individuals vs married couple affects the overall survival of patients diagnosed with GBM.


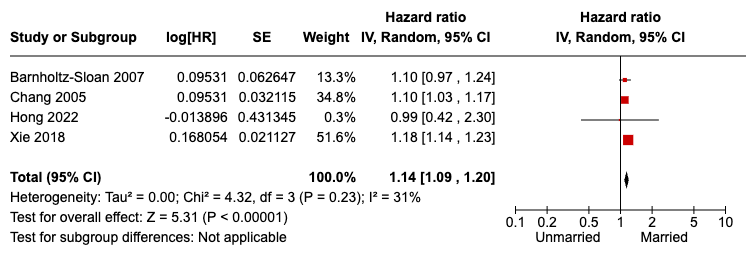

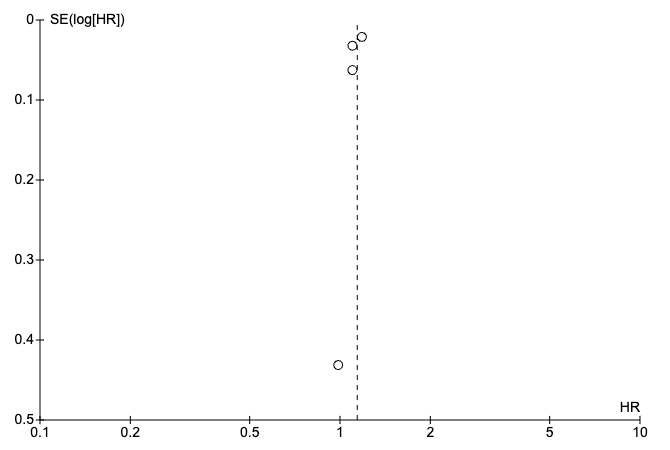


**Supplementary file 6.** A meta-analysis investigating widowhood vs married couple affects the overall survival of patients diagnosed with GBM.


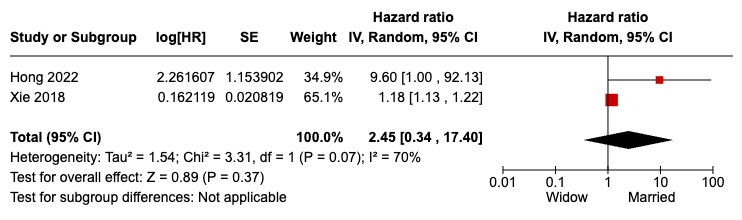

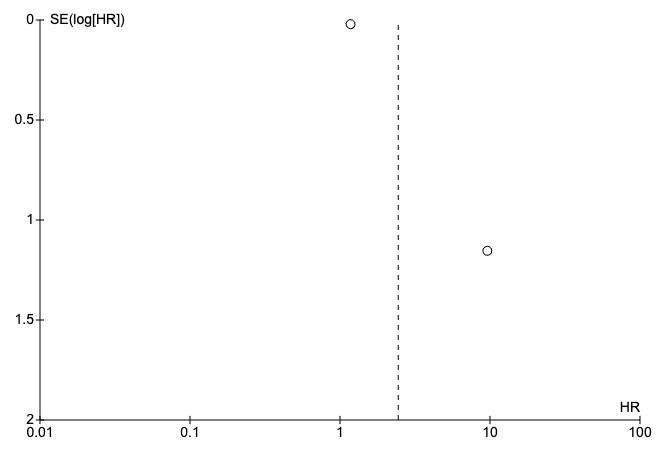


**Supplementary file 7.** A meta-analysis investigating of unknown marital status vs not married individuals affects the overall survival of patients diagnosed with GBM.


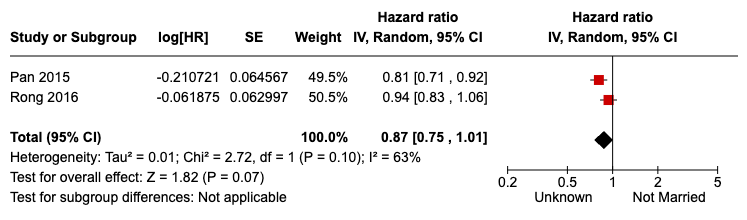

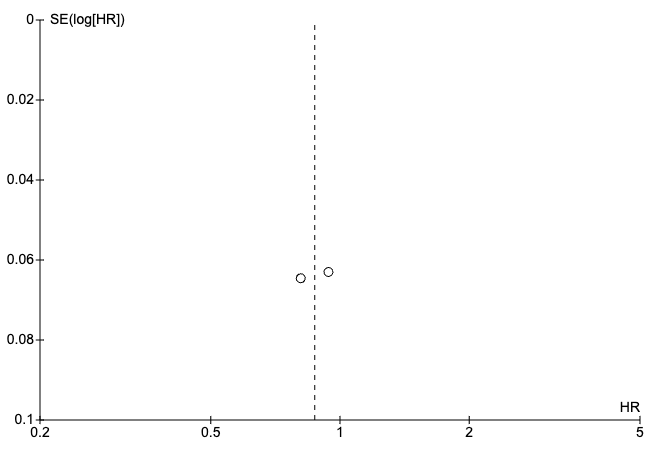


**Supplementary file 8.** A meta-analysis investigating private payer vs private insurance affects the overall survival of patients diagnosed with GBM.


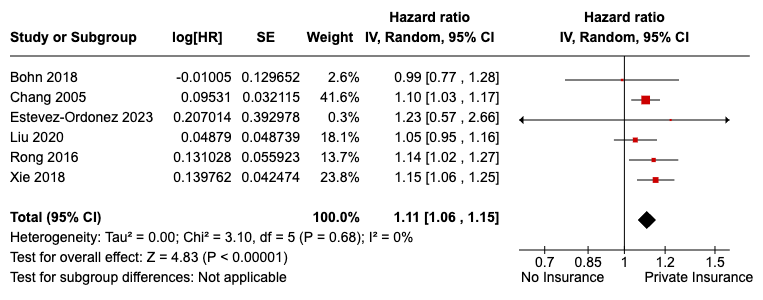


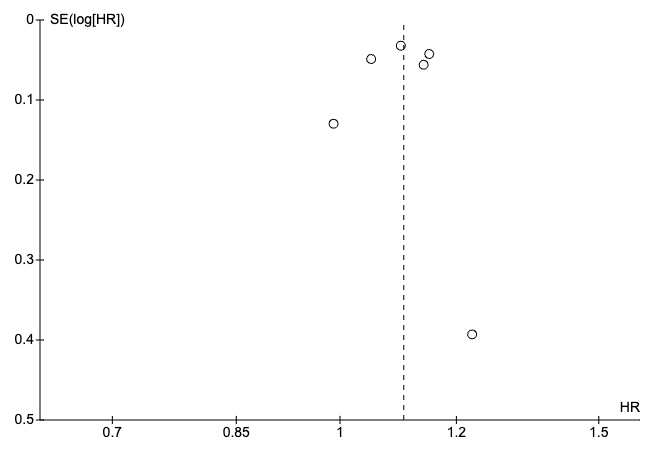


**Supplementary file 9.** A meta-analysis investigating government based insurance vs private insurance affects the overall survival of patients diagnosed with GBM.

**
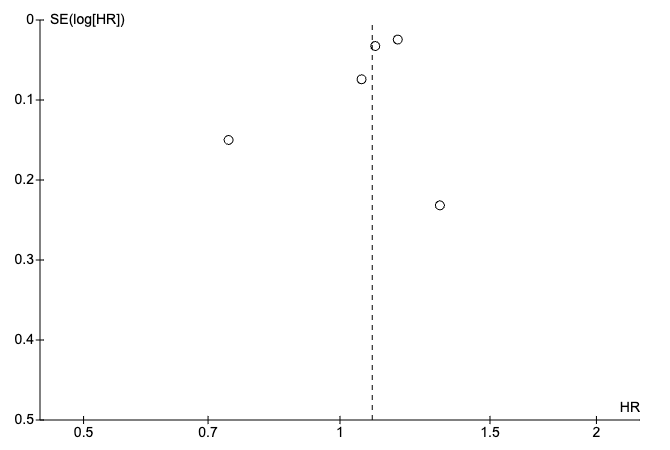

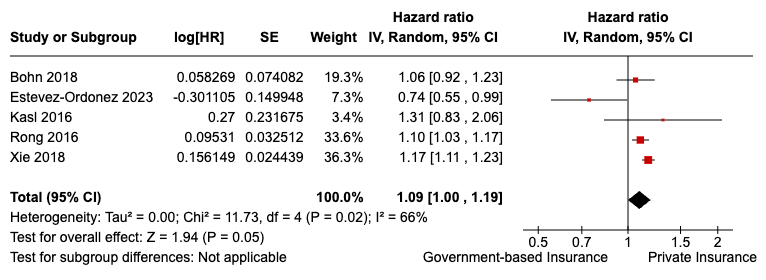
**

**Supplementary files 10** A meta-analysis examining the influence of concurrent medical conditions on the overall survival rates among patients diagnosed with GBM.


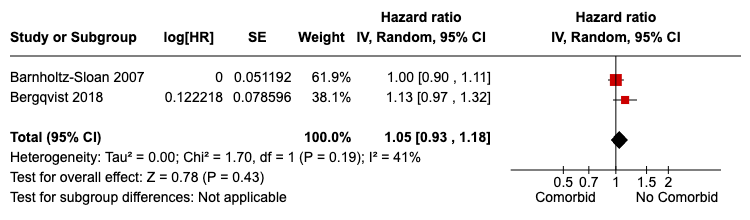

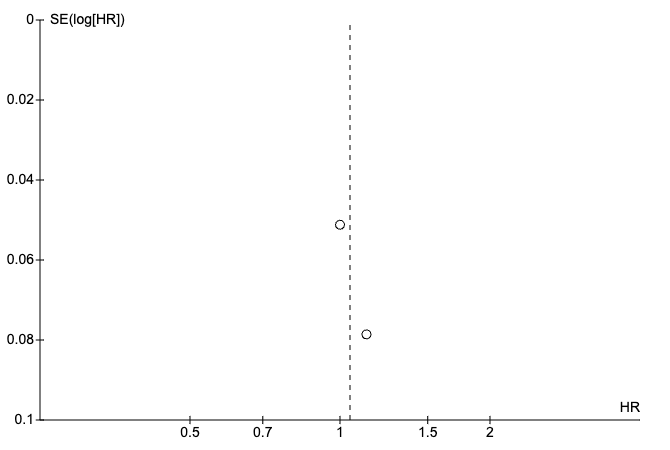


**Supplementary files 11** A meta-analysis examining the influence of low income vs high income on the overall survival rates among patients diagnosed with GBM.


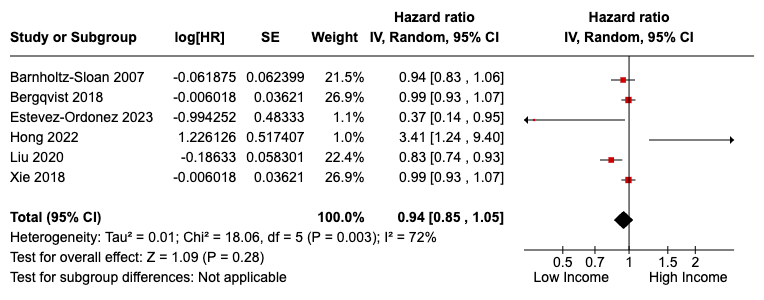


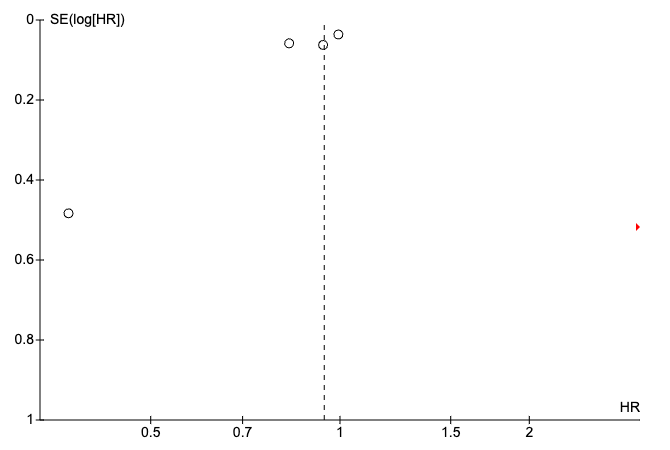


**Supplementary files 12** A meta-analysis examining the influence of urban vs metropolitan on the overall survival rates among patients diagnosed with GBM.

**
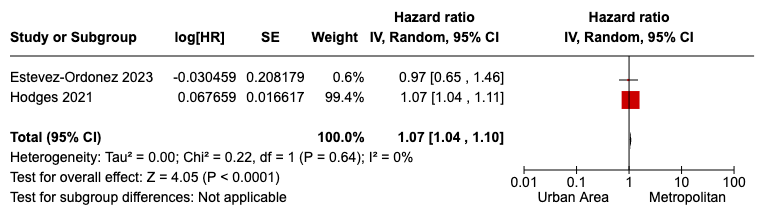

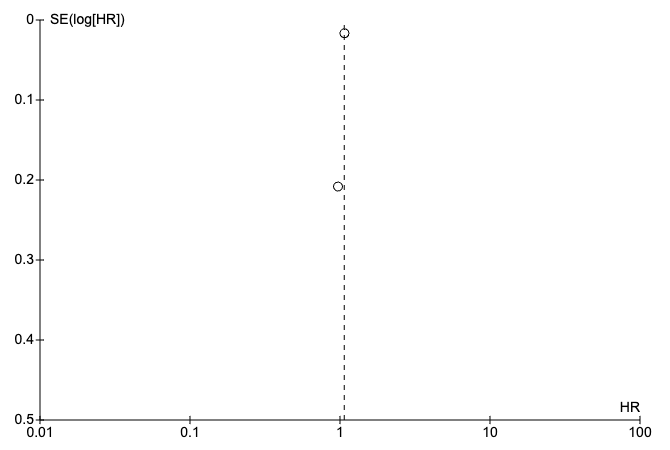
**

**Supplementary files 13** A meta-analysis examining the influence of rural vs metropolitan on the overall survival rates among patients diagnosed with GBM.


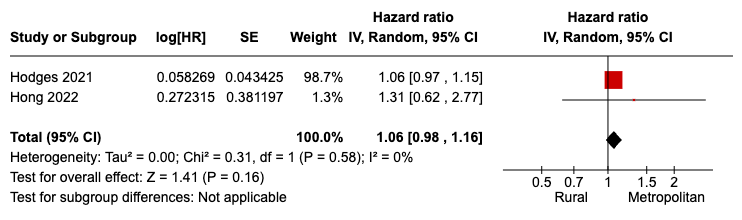

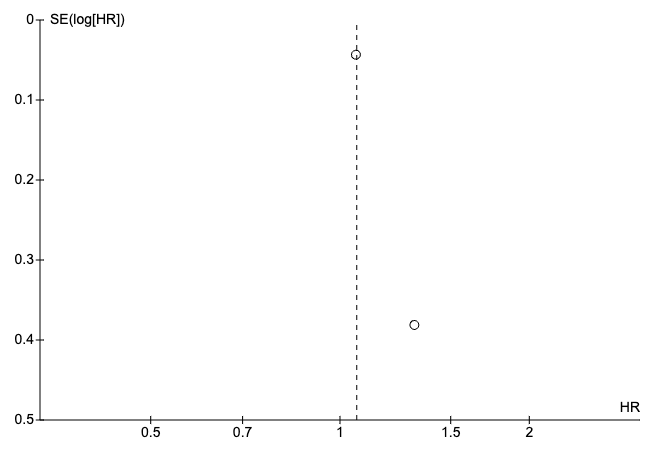

Supplement: Supplementary file 1 — Supplementary Material 1 [file 10143_2025_3647_MOESM1_ESM.docx]
